# The Mosquito—a Cog in the Ideal Nature Machine

**DOI:** 10.3201/eid2105.AC2105

**Published:** 2015-05

**Authors:** Carmen C.H. Petrosian-Husa

**Affiliations:** Independent anthropologist and art historian, Vienna, Austria

**Keywords:** emerging infectious diseases, art science connection, the mosquito, a cog in the ideal nature machine, ideal nature machine, installation, Stefan Waibel, modern art, installation, mosquito, nature, about the cover

**Figure:**
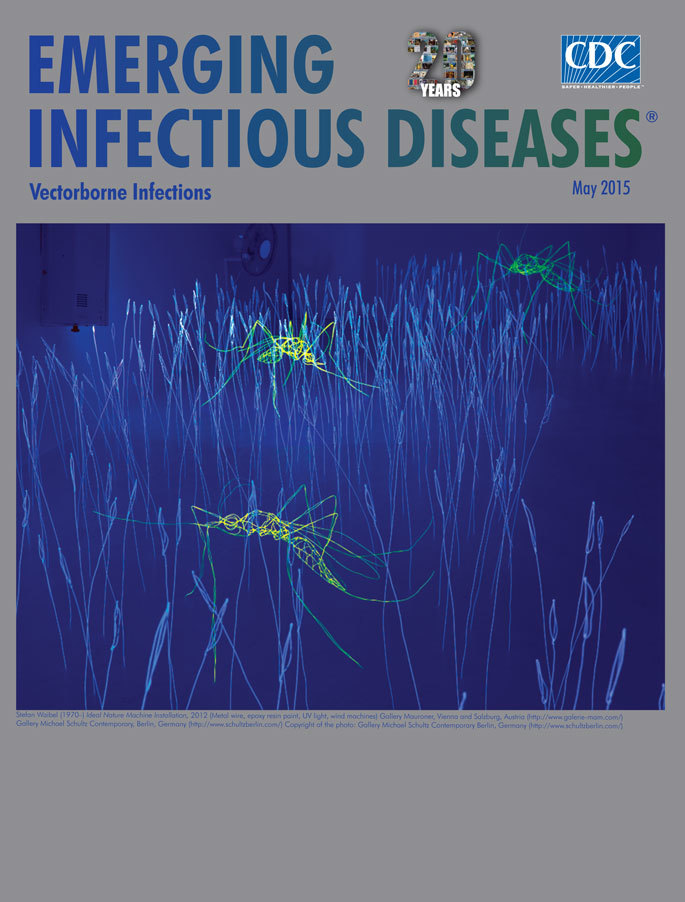
**Stefan Waibel (1970–) *Ideal Nature Machine.* Installation, (metal wire, epoxy resin paint, UV light, wind machines 2012).** Gallery Mauroner Vienna and Salzburg, Austria (http://www.galerie-mam.com/); Gallery Michael Schultz Contemporary Berlin, Germany (http://www.schultzberlin.com/)

“You stagger as well as you may.
Your own imponderable weightlessness
Saves you, wafts you away on the very draft my anger makes in its snatching.”
—The Mosquito by D.H. Lawrence

The installation *Ideal Nature Machine* by Austrian artist Stefan Waibel is a minimalistic depiction of a piece of turf where several mosquitoes are resting among the grass blades. The installation can be adjusted to fit different rooms and can measure from 5 to 350 square meters depending on the venue. Its presentation in a dark setting that is illuminated by UV light emphasizes the work’s artificiality; wind machines, which cause the grass blades and the mosquitoes to slightly sway, make the work seem more lifelike. (Installations built for an outdoor presentation are made of sturdier materials than are those built for indoor exhibition and do not use the wind machine because the grass blades and mosquitoes are naturally exposed to wind and weather.)

The noise from the wind machines adds to the illusion of a hot night, when the whizzing sound of a single mosquito can escalate from awareness to annoyance. The dark surroundings paired with the intensive colors created by the UV light suggest the sweltering atmosphere of a tropical night, the kind of night when a layer of heat and humidity covers everything and a fan stirring the air cannot prevent beads of perspiration from developing on the skin. This installation, thus, evokes not only nature but also those natural sensations.

The grass blades are minimalistic but measure about half a meter, so they are just as tall as those in an unmown meadow. The mosquitoes, though, measure ≈1.5 m and are 300 times as large as the actual average (5 mm) insect. In *Ideal Nature Machine*, Waibel does not depict any other animals, just the mosquitoes, but he invites the visitors to participate in his installations, to step in and walk around, and in this way to become part of it.

As viewers, we do not know among what kind of grass blades we are walking, nor do we know what kind of mosquitoes are hiding there. Perhaps these mosquitoes are resting, which they do for a large portion of their gonotrophic cycle (blood feeding, egg maturation, and oviposition, a cycle which is repeated several times throughout the life of adult females). These are likely exophylic mosquitoes, spending this resting period outside human dwellings, usually preferring shady areas such as among tall grass blades.

Nature may be viewed as an ideal, but multifaceted machine that functions without concern for consequences. Mosquitoes are an efficient and even graceful component; they provide food for bats and other insectivorous animals. Some mosquitoes are effective vectors that contribute to the spread of disease-causing agents that have serious and widespread consequences for humans. Mosquitoes transmit five species of *Plasmodium* parasites that cause malaria in humans and a constellation of infectious agents that can cause yellow fever, West Nile virus disease, chikungunya virus infection, Rift Valley fever, Japanese encephalitis, lymphatic filariasis, and dengue hemorrhagic fever.

Stefan Waibel regards mosquitoes as the plankton of the air, thus, codifying their abundance as a cog within nature’s and the machine’s cogwheels. Waibel’s series of Ideal Nature Machines installations underscore that the “Machine Nature” would not work without its myriad cogs and components, mosquitoes included. Efforts to track, study, and prevent diseases by controlling mosquitoes and other vectors, as described in many of this issue’s articles, also underscore the complex, intertwined relationships that fuel creative thought in both art and science.
